# Investigating the Correlation between the Microstructure and Electrical Properties of FeSbO_4_ Ceramics

**DOI:** 10.3390/ma15196555

**Published:** 2022-09-21

**Authors:** Carlos G. P. Moraes, Robert S. Matos, Cledson dos Santos, Ştefan Ţălu, John M. Attah-Baah, Romualdo S. Silva Junior, Marcelo S. da Silva, Marcos V. S. Rezende, Ronaldo S. Silva, Nilson S. Ferreira

**Affiliations:** 1 Department of Physics, Federal University of Sergipe, São Cristovão 49100-000, Brazil; 2 Laboratory of Corrosion and Nanotechnology (LCNT), Federal University of Sergipe, São Cristovão 49100-000, Brazil; 3 Amazonian Materials Group, Federal University of Amapá, Macapá 68911-477, Brazil; 4 The Directorate of Research, Development and Innovation Management (DMCDI), Technical University of Cluj-Napoca, 15 Constantin Daicoviciu St., 400020 Cluj-Napoca, Romania; 5 Federal Institute of Education, Science and Technology of Sertão Pernambucano, Salgueiro 56000-000, Brazil

**Keywords:** iron antimonate, FeSbO_4_, powder, ceramics

## Abstract

FeSbO_4_ powder was prepared using the solid-state reaction method in this work. Afterward, the dense and porous ceramics were obtained by sintering the pressed powder calcined at temperatures of 900 and 1000 °C for 4 h. Rietveld profile analysis of the X-ray powder diffraction data showed that FeSbO_4_ adopts the trirutile-type structure (space group *P*4_2_/*mnm*, with a ≅ 4.63 Å and c ≅ 9.23 Å). SEM images showed that the powder calcined at 900 °C after being sintered at 1200 °C resulted in ceramics of higher crystallinity, larger grains, and consequently, low porosity. The dielectric properties were measured in the frequency range of 10^−1^ Hz–1 MHz as a function of temperature (25–250 °C). The real (σ′) and imaginary (σ″) parts of the complex conductivity increase with rising annealing temperature for both samples. The real conductivity in the AC region for 𝑓 = 100 kHz was $1.59\times 10^{-6}\;S\cdot {\mathrm{cm}}^{-1}$ and $7.04\times 10^{-7}\;S\cdot {\mathrm{cm}}^{-1}$ for the ceramic samples obtained from the powder calcined at 900 (C-900) and 1000 °C (C-1000), respectively. Furthermore, the dielectric constants (k′) measured at room temperature and f=100 kHz were 13.77 (C-900) and 6.27 (C-1000), while the activation energies of the grain region were E_a_ = 0.53 eV and E_a_ = 0.49 eV, respectively. Similar activation energy (E_a_ = 0.52 eV and 0.49 eV) was also obtained by the brick-layer model and confirmed by the adjustment of activation energy by DC measurements which indicated an absence of the porosity influence on the parameter. Additionally, loss factor values were obtained to be equal to 3.8 (C-900) and 5.99 (C-1000) for measurements performed at 100 Hz, suggesting a contribution of the conductivity originated from the combination or accommodation of the pores in the grain boundary region. Our results prove that the microstructural factors that play a critical role in the electrical and dielectric properties are the average grain size and the porosity interspersed with the grain boundary region.

## 1. Introduction

The family of ceramic materials with ABO_4_-type structures have been investigated since the last century due to their promising applications, such as photodetector system, solar energy conversion, gas sensor, photocatalyst, and electrode material for Li-ion batteries, etc. [1,2,3,4,5,6,7]. In this context, the compound belonging to this rutile-type ABO_4_ family, called iron antimonate FeSbO_4_, has been the subject of several researchers because it is a potential candidate for several applications, such as gas detector sensors and catalysis properties. This particular compound has been widely investigated for more than 100 years after its first mineral description; however, its crystal structure (rutile or trirutile types) is still a long-standing controversial debate in the literature [8]. FeSbO_4_ has been reported to crystallize in two different forms: a rutile-type (space group *P*4_2_/*mnm* with a ≅ 4.63 Å and c ≅ 3.07 Å) [9] and a superstructure of rutile with a tripling along the axis of the rutile structure base (c’ = 3c) corresponding to the trirutile-type structure (space group *P*4_2_/*mnm* with a ≅ 4.63 Å and c ≅ 9.23 Å) [10]. Despite several reports on FeSbO_4_, most studies have focused mainly on its catalytic properties [11,12,13]. Moreover, there are few experimental studies on the physical properties of FeSbO4, such as magnetic and structural properties [5,14,15,16,17,18], although research efforts towards their electrical properties are still scant. For instance, Millet et al. [3] studied the electrical conductivity of FeSbO_4_ and reported on the n-type semiconductor behavior of this compound. They also reported that a surface transformation by forming antimony oxide and a rutile-type solid solution with only cationic vacancies is likely responsible for changes in the type of electrical conductivity of VSbO_4_-FeSbO_4_. Recently, Singh et al. [4] reported that the electrical resistance of FeSbO_4_ films is quite dependent on the temperature. Their analysis confirmed that lower temperature induces high resistance values which tend to decrease with further temperature increases. This behavior was attributed to a resistance-altering effect based on oxygen surface species and surface coverage. Additionally, they found an activation energy value of 0.49 eV and a maximum sensitivity of 2.9 MΩ∙s^−1^ for the film, which was about 12 times greater than that obtained for the not sintered pellet powder. Nevertheless, to the best of our knowledge, studies on the electrical and dielectric properties of sintered FeSbO_4_ ceramics have not yet been conducted.

Here, we report on the solid-state synthesis of FeSbO_4_ powder and after, the obtention of dense ceramics by sintering the pressed powder at temperatures of 900 and 1000 °C for 4 h. The present work also aims for the first time to clarify the role of microstructures (such as pellet densification and grain growth) on the electrical and dielectric properties of FeSbO_4_ ceramics through a combination of electron microscopy, EDS, and XRD characterizations.

## 2. Materials and Methods

The FeSbO_4_ powder was prepared by a solid-state reaction technique using high-purity Sb_2_O_3_ (Êxodo Scientifica, 99.5%, Sumaré, Brazil) and α-Fe_2_O_3_ (Sigma Aldrich, 96%, Saint Louis, USA) in stoichiometric ratio. In a typical procedure, the oxides were first mixed with agate mortar and calcined at optimum temperatures of 900 °C (C-900) and 1000 °C (C-1000) for 4 h to obtain the FeSbO_4_ phase. The powder formation and purity were first checked by X-ray diffraction (XRD) analysis before the ceramic-pressing process. Such XRD analysis has been performed using a Rigaku DMAX Ultima+ diffractometer with monochromatic Cu-Kα radiation (γ = 0.154056 nm) at 40 kV and 40 mA in angular 2*θ* range from 20° to 80° with 0.02° step size. The calcination process and XRD analysis were repeated until a single compound phase was formed. The lattice parameters and atomic coordinates have been obtained via Rietveld refinement using the FullProf Suite of programs [19]. Afterward, an appropriate amount of each calcined powder was mixed with a small amount of polyvinyl alcohol (PVA) which were then grounded and pressed into cylindrical pellets forms of 4 mm diameter and 1–2 mm thickness by using a hydraulic press at uniaxial pressure of 9.81 × 10^6^ N∙m^−2^. The ceramic pellets were sintered at a temperature of 1200 °C for 4 h.

The surface morphology, degree of agglomeration, and average grain size of the FeSbO_4_ powder and ceramic bodies were obtained by scanning electron microscopy (SEM-EDS JEOL-JSM6510LV). Qualitative chemical analyses were done using energy-dispersive X-ray spectroscopy (EDX). The surface of the ceramic bodies did not undergo any polishing process before the measurements.

The average of the sintered ceramic particles was measured directly on the micrograph, considering the larger dimension of all image particles. For the interposed ceramics, the average grain diameter was determined using the intercept method, according to ASTM (American Society for Testing Materials) E112 standard [20]. In this procedure, four lines are randomly drawn on two SEM images of each sample, recording the number of grain boundaries intercepted by each line, as shown in Equation (1):

(1) $$\underline{D}=1.56\;\underline{L}$$

where $\underline{D}$ is the average total grain size, $\underline{L}$ is the ratio between the length of the lines (according to the micrograph scale) and the number of intercepted contours, and the value 1.56 is a parameter shape [21].

The apparent density measurements of the sintered ceramic bodies were obtained by the Archimedes method, according to the NBR-6220 standard of ABNT (Brazilian Association of Technical Standards), utilizing Equations (2) and (3), as follows:

(2) $$d_{apparent}=\frac{m_{s}}{\left(m_{u}-m_{sub}\right)}d_{H_{2}O}$$

where $m_{s}$ is the dry mass, $m_{u}$ is the wet mass, and $m_{sub}$ is the submerged mass of the ceramic body. Furthermore, the porosity, *P*, was obtained from Equation (3):

(3) $$P=\left(1-\frac{d_{apparent}}{d_{theoretical}}\right)\times 100\%$$

where $d_{theoretical}$ is the density of the pure single crystal of FeSbO_4_.

The ceramic pellets were made smooth by polishing their faces with SiC sandpaper (1200 and 2000). Afterward, the surfaces were made into conducting surfaces by coating them with silver paste. Before the electrical characterization process, the ceramic samples were cleaned with isopropyl alcohol in an ultrasonic vat for 2 min, receiving a layer of silver paint and finishing with heating at 300 °C for 2 h to remove the solvent and crystallize the silver electrodes. A Solartron phase/gain/impedance analyzer 1260 and an additional Solartron 1296 Dielectric Interface (Solartron Analytical, Leicester, England) were used in impedance spectroscopy measurements on FeSbO_4_ ceramics in a temperature range between 25 °C to 250 °C. The applied voltage shows a profile of the type $V\left(t\right)=V_{0}e^{-i\omega t}$ with amplitude *V*_0_ = 100 mV, and scans were performed from 0.1 Hz to 1 MHz for data acquisition through the Smart Software [22]. The temperature variation on the sample was employed by an electrical furnace with Eurotherm 2216 external controller. The Zview program [23] adjusted the data using the brick-layer model.

## 3. Results and Discussion

**Figure 1:**
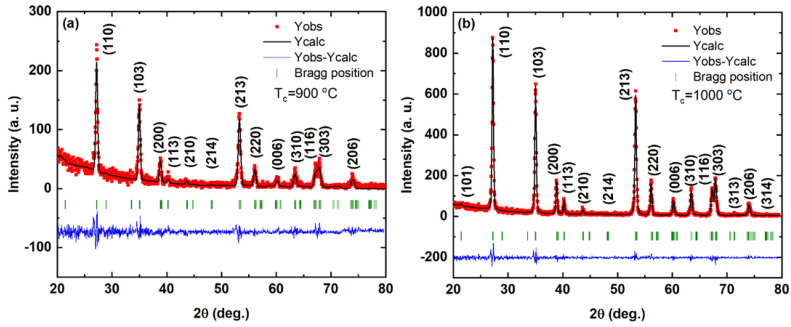
The observed (red squares), calculated (solid line), and difference X-ray powder diffraction patterns obtained from a Rietveld refinement for the FeSbO_4_ powder calcinated at: (**a**) 900 °C and (**b**) 1000 °C. The green vertical ticks indicate the calculated Bragg peaks allowed by the space group *P*4_2_/*mnm* (#136).

First, we discuss the crystal structure which will be a piece of information necessary for further discussion on the electrical and dielectric behavior of FeSbO_4_. Herein, the powder X-ray diffraction pattern of FeSbO_4_ was analyzed by conventional Rietveld refinement, as shown in Figure 1. No trace of possible impurity from any secondary phase or unreacted precursor was observed. For the profile refinement, we assumed equal displacement factors for all atoms and a Thompson–Cox–Hastings profile of the reflections. The background was constructed by superposing Chebychev polynomials of a higher degree. A first attempt to refine the XRD patterns was made by imposing Fe^3+^ and Sb^5+^ randomly distributed in the 2a site and O occupying the 4f site and assuming the rutile-type structure (space group *P*4_2_/*mnm* with a ≅ 4.63 Å and c ≅ 3.07 Å), which resulted in a reasonably high R_wp_ value (6.4%). However, the refinement readily converged to Bragg R-factors of ~2.6–2.9% (Rf-factor ~2.7–3.0%) as we assumed a trirutile-type (space group *P*4_2_/*mnm* with a ≅ 4.63 Å and c ≅ 9.23 Å) with positional parameters such as Sb^5+^ (at *z* = y = z = 0 and *z* = y = z = 1/2) and Fe^3+^ (at *z* = y = 0, z = 2/3, and *z* = y = ½, z = 1/6), and equal probability distributed for Sb^5+^ and Fe^3+^ on (b) (at *z* = y = 0, z = 1/3) and (at *z* = y = 1/2, z = 5/6) sites. These metal sites are surrounded by O^2−^ octahedra, as reported for FeSbO_4_ by Berry et al. [10]. Then, we conclude by comparing both Rietveld refinements that the synthetic FeSbO_4_ crystallizes in a superstructure of rutile, corresponding to a tripling along the axis of the rutile structure base (c’ = 3c) unit cell in which Fe^3+^ ions are arranged in square planar layers separated by neighboring double layers of edge-connected SbO_6_ octahedra. The refined structural parameters are summarized in Table 1.

The X-ray density steadily decreases from ~6.1 to ~5.6 g∙cm^−3^ with increasing powder calcination temperatures from 900 to 1000 °C, which is in good agreement with the density value of 5.82 g∙cm^−3^ reported for the FeSbO_4_ [24]. The apparent densities and porosities of the ceramic bodies have also been calculated using the Archimedes immersion method. The highest relative density of (98.9 ± 0.2)% and lowest porosity of (1.1 ± 0.2)% were obtained for the C-900 ceramics pellets. However, the C-1000 ceramic exhibited the lowest relative density (68.2 ± 0.1)% and highest porosity (36.8 ± 0.1)%. Higher relative density and low porosity for C-900 ceramic were likely due to the smaller grain size of the calcined powder used for producing this ceramic. This indicates that the mean grain size range for the C-900 powder has a great influence on the kinetics of the sintering process.

**Figure 2:**
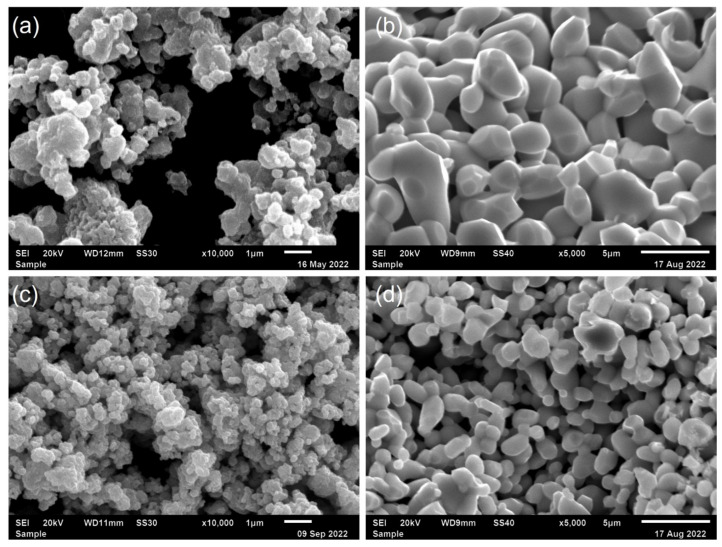
SEM images for FeSbO_4_ powder calcined at (**a**) 900 °C and (**c**) 1000 °C for 4 h and fractured cross-sections of FeSbO_4_ pellets, (**b**) C-900 and (**d**) C-1000, sintered at 1200 °C for 4 h.

**Figure 3:**
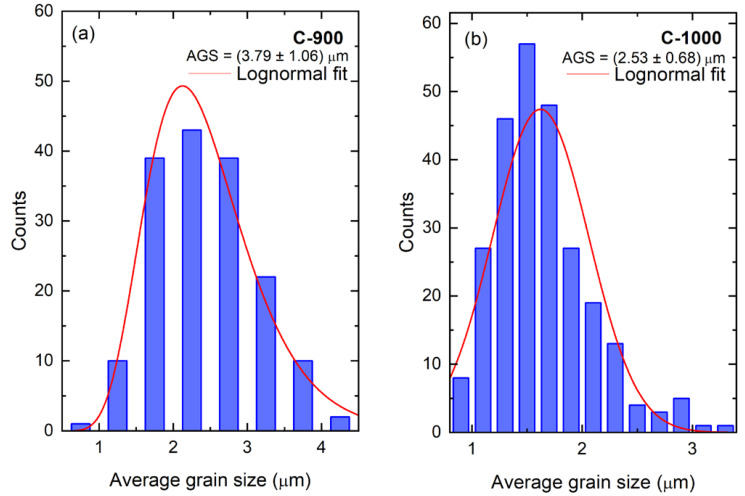
The average grain size of ceramics: (**a**) C-900 and (**b**) C-1000.

The microstructures of the FeSbO_4_ powder and sintered ceramics samples are presented in Figure 2. It is clearly observed from the SEM micrographs, shown in Figure 2a,c, that the grains have large lumps with irregular shapes and considerable agglomeration. It is found through high magnification that the agglomerates consist of primary particles with the size of (0.73 ± 0.51) μm (P-900) and (1.67 ± 0.54) μm (P-1000). Figure 2b,d provide a summary of the SEM images of the fractured cross sections for the FeSbO_4_ ceramic pallets sintered C-900 and C-1000, respectively. The grains have elongated and spherical structures, most likely associated with an open crystal structure of FeSbO_4_. This structure contains two-dimensional layers of three-edge shared SbO_6_ octahedra, which appear like an open honeycomb with holes, whereas FeO_6_ octahedra connect each layer near holes, favoring low surface energy. We have also analyzed the micrographs of Figure 2b,d using the intercept method, according to the ASTM E112 standard. The particle size distribution of the powder is given in Figure 3a,b, which shows that C-900 ceramic pellet grain appears larger (~3.8 μm) and connected, resulting in a nearly fully dense ceramic with lower residual pores, which correlates with its high relative density (~99%) obtained by the Archimedes method. In contrast, the C-1000 ceramic shows smaller grains (~2.5 μm) and a low degree of sintering with a significant degree of porosity following the previously discussed result of ~39%. The higher interconnection grains number with clusters of small grains forming densely packed aggregates (2.55 ± 1.25) μm can also justify this significant porosity, as observed in the SEM image (Figure 2d). Thus, we can see that the initial powder particle size distribution significantly affects the density and microstructure of the ceramics, as reported in other oxides [25,26,27]. Herein, the larger particle sizes for the P-1000 powder resulted in ceramics with higher porosities than those produced using the P-900 powder with smaller particles. In fact, the higher surface energy for smaller particle sizes promotes a higher sintering rate of the compacted powder, resulting in an early packing process by favoring the dense aggregates to grow quickly, forming more prominent grains, and then inducing a significant decrease in the pore volume.

The EDS analysis reveals the existence of constituent elements, Fe, Sb, and O, confirming the purity of the FeSbO_4_ powder and ceramics. For the C-900 sample, the actual atomic percentage was 21.1 ± 0.2 (Fe), 19.0 ± 0.2 (Sb), and 59.9 ± 1.3 (O) at.%, while 19.8 ± 0.3 (Fe), 17.3 ± 0.1 (Sb), and 62.9 ± 0.7 (O) at.% were observed for the C-1000 sample. Furthermore, these results also agree with the chemical composition of FeSbO_4_ ceramics grains, whereas Fe:Sb atomic % ratios of (18.4 ± 0.2):(18.9 ± 0.1) and (19.6 ± 0.2):(17.6 ± 0.1) in the C-900 and C-1000 samples, respectively, are in the range of ideal tripuhyite (Fe:Sb = 1:1) [15,16].

**Figure 4:**
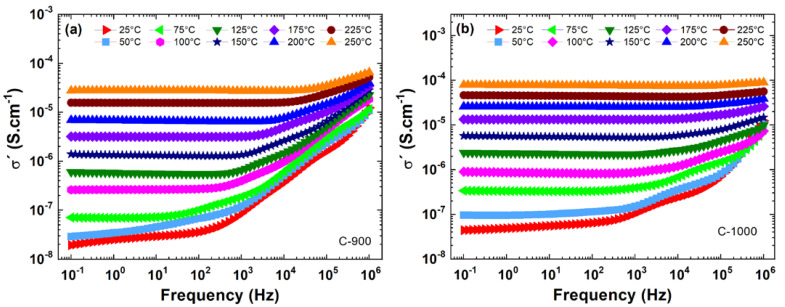
Results of the real electrical conductivity ($\sigma^{\prime }$) versus frequency (f) for ceramic pellets sintered at (**a**) C-900; (**b**) C-1000. Electrical measurements were performed in isotherms from 25 to 250 °C.

Figure 4 shows the real part ($\sigma^{\prime }$) of the complex electrical conductivity ($\sigma^{*}=\sigma^{\prime }+i\sigma^{\prime \prime }$) versus frequency with varying temperatures from 25 to 250 °C for ceramic pellets uniaxially pressed at $100\;\mathrm{kgF}\cdot {\mathrm{cm}}^{-2}$ and sintered at 1200 °C for 4 h. Analyzing the alternating current (AC) conductivity results in Figure 4, both samples of FeSbO_4_ (C-900 and C-1000) showed dispersion of conductivity, $\sigma^{\prime }$, at high frequencies and dependence on temperature. In the direct current (DC) region with low frequencies (grain boundary region—GB) and T=25 °C, the sample C-1000 showed a higher value in its conductivity in relation to the sample C-900. Both samples reveal a dependence with $\omega^{n}$ according to Jeep Dyre’s model [28], where n varies between 0.6 to 1.0 at high frequencies.

**Figure 5:**
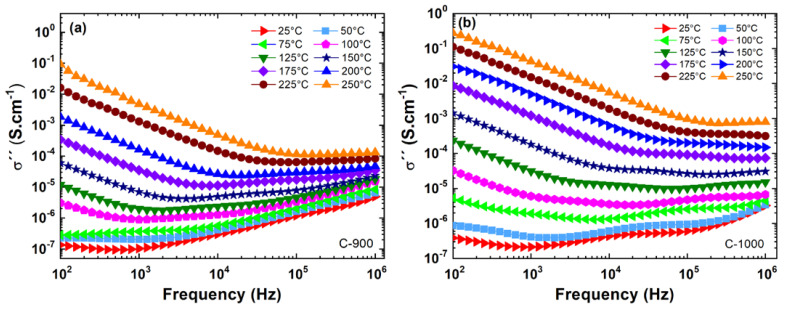
Results of the imaginary electrical conductivity ($\sigma^{\prime \prime }$) versus frequency (f) for ceramic pellets sintered at (**a**) C-900; (**b**) C-1000. Electrical measurements were performed in isotherms from 25 to 250 °C.

The C-900 sample with AGS=3.79 μm showed a smaller dispersion in its imaginary conductivity ($\sigma^{\prime \prime }$) at low frequencies compared to sample C-1000 $\left(2.53µm\right)$, according to the results in Figure 5. At high temperatures, both samples showed excessive noise in the region between 0.1 to 10 Hz. For this reason, these measurements in this frequency range were omitted in Figure 5. This problem is associated with noise in the sample holder electrodes in measurements performed at high temperatures.

**Figure 6:**
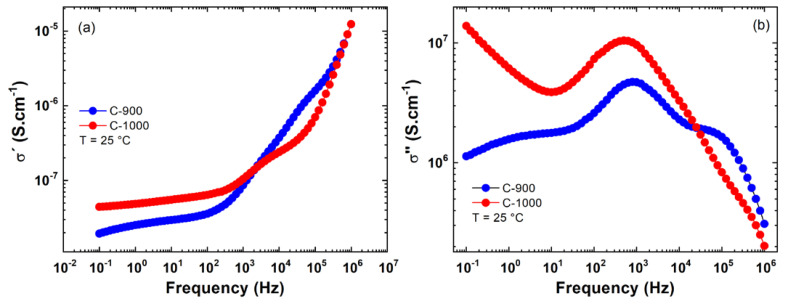
Results of the electrical conductivity versus frequency for C-900 and C-1000 ceramics: (**a**) real part ($\sigma^{\prime }$); (**b**) imaginary part ($\sigma^{\prime \prime }$).

In Figure 6a,b the real ($\sigma^{\prime }$) and imaginary ($\sigma^{\prime \prime }$) conductivities are presented as a function of frequency, f, performed at 25 °C. Analyzing the AC region for 𝑓 = 100 kHz, considering a grain contribution, sample C-1000 showed a lower conductivity than C-900; see Table 2. Compared with the average grain size results obtained via SEM measurements (see Figure 2), it can probably be associated with grain size. In other words, the small grains produce lower electrical conductivity, $\sigma^{\prime }$. Millet et al. [3] obtained a DC conductivity value of $6.30\cdot 10^{-9}S\cdot {\mathrm{cm}}^{-1}$ for a FeSbO_4_ green body pellet pressed at $10^{5}$ Pa at room temperature (approximate value obtained graphically). However, at f=10 Hz, a substantial increase, around 1.9 times, in the ac electrical conductivity of the ceramic sample is observed, which has a P=36.8% (C-1000) compared to the sample with P=1.1% (C-900); see Table 2.

According to Peko et al. [29], certain concentrations of porosities possibly lodged in the grain boundary region favor an increase in this conductive region associated with a significant drop in the corresponding activation energy.

**Figure 7:**
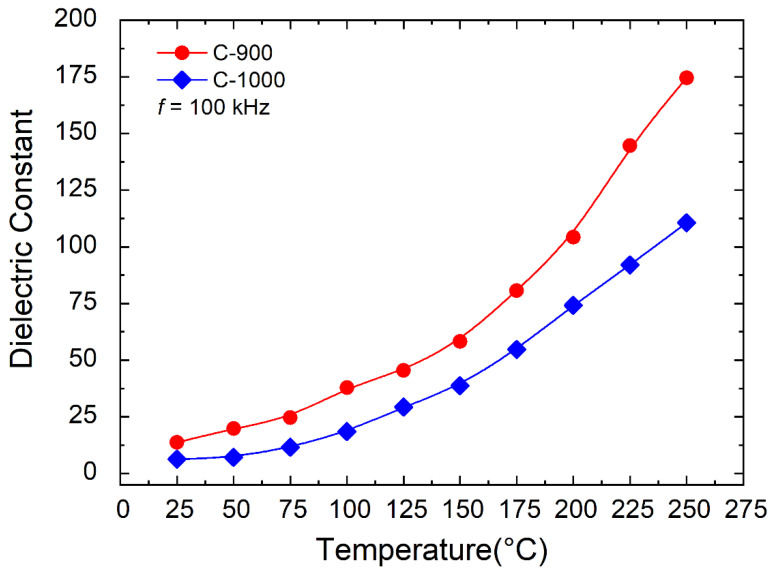
The temperature-dependent dielectric constant of frequency for C-900 and C-1000 ceramics.

Figure 7 shows the frequency dependence of the room temperature dielectric constant of FeSbO_4_ ceramics C-900 and C-1000 with 𝑓 = 100 kHz. The real dielectric constant was obtained from the capacitance data as a function of frequency, performed by the FRA Solatron 1260/Dielectric Bridge 1296. To convert the data from C to real dielectric constant, Equation (4) was used:

(4) $$\kappa^{\prime }=\frac{\varepsilon^{\prime }}{\varepsilon_{0}}=\frac{C^{\prime }\cdot d}{\varepsilon_{0}A}$$

where $\varepsilon^{\prime }$ is the actual electrical permittivity (𝐹∙𝑚^−1^), $C^{\prime }$ is the capacitance (F), *𝑑* is the measurement cell thickness in 𝑚, 𝐴 is the area of the cylindrical face of the ceramic sample in *m*^2^, and $\varepsilon_{0}=8.8542\times 10^{-12}\;F\cdot m^{-1}$.

Table 2 shows the results for the dielectric constant of the FeSbO_4_ ceramic samples sintered at 1200 °C for 4 h at 𝑇 = 25 °C and 𝑓 = 100 kHz. According to Table 2, the FeSbO_4_ ceramic sample C-900 presented a dielectric constant 219.6% higher than the sample C-1000. All ceramics showed temperature-dependent dispersion in the dielectric constant and that this increase occurs at higher temperatures (Figure 7). The fact that the dielectric constant was measured at high frequencies at *f* = 100 kHz means that this increase in this magnitude, $\kappa^{\prime }$, is possibly due to the increase in the average grain size and not due to the increase in porosity, P.

The complex impedance measurements, $Z^{\prime }$ and $Z^{\prime \prime }$, as a function of frequency with varying temperature were performed by applying an electrical potential of 100 mV and in a frequency range of $10^{-1}$ to $1\times 10^{6}\;\mathrm{Hz}$. The measurement method used was the two-probe technique. The resistivity results ($\rho^{\prime }$ and $\rho^{\prime \prime }$) in the complex plane were obtained from the normalization of the impedance data ($Z^{\prime }$ and $Z^{\prime \prime }$) through the geometric parameters of the sample:

$$\frac{A}{d}=\frac{Cross-sectional\;area\;of\;cylindrical\;}{thickness}$$

given by the following Equations (5) and (6):

(5) $$\rho^{\prime }=Z^{\prime }\frac{A}{d}$$

(6) $$\rho^{\prime \prime }=Z^{\prime \prime }\frac{A}{d}$$

Several analysis models based on equivalent circuits are found in the literature, which is applied in different types of systems, especially in ceramics. In ceramic systems, where the relaxation frequency of the electrical impedance of the grain boundary is distinguishable from that of the grain, the most used equivalent circuit consists of two parallel resistor-capacitor blocks (RC) connected in series with each other. Among these analysis models that use equivalent circuits, the brick-layer model, so called because it treats the microstructure as an array of cubic grains separated by flat grain boundaries, is one of the most used for analyzing the electrical and dielectric properties of ceramic materials. The brick-layer model presupposes fine, continuous, and highly resistive grain boundaries, which would result in a one-dimensional current flow through the grain and transverse to the contour [30,31]. The complex electrical impedance associated with the parallel RC circuit of each region is described by Equation (7):

(7) $$Z^{*}\left(\omega \right)=\frac{R}{\left(1+i\omega \mathrm{RC}\right)}$$

Multiplying the conjugate term $\left(1-i\omega \mathrm{RC}\right)$ on both terms of Equation (7) and then separating the contribution from the real and imaginary parts of the complex impedance leads to Equation (8):

(8) $$Z^{*}\left(\omega \right)=\frac{R}{\left[1+{\left(\omega \tau \right)}^{2}\right]}-i\frac{R\omega \tau }{\left[1+{\left(\omega \tau \right)}^{2}\right]}=Z^{\prime }-iZ^{\prime \prime }$$

where the constant τ=RC is the relaxation time of the circuit and the real ($Z^{\prime })$ and imaginary $(Z^{\prime \prime })\;$components of the complex impedance can be defined by Equations (9) and (10):

(9) $$Z^{\prime }=\frac{R}{\left[1+{\left(\omega \tau \right)}^{2}\right]}$$

and

(10) $$Z^{\prime \prime }=\frac{R\omega \tau }{\left[1+{\left(\omega \tau \right)}^{2}\right]}$$

However, experimentally, impedance spectroscopy diagrams do not always present semicircles centered on the real axis. This decentralization results from the existence of a distribution of relaxation times, $\omega_{0}\tau =1$ and τ=RC, rather than a single value. In this case, an empirical correction is made in Equation (7) which takes the form shown in Equation (11) [31]:

(11) $$Z^{*}\left(\omega \right)=\frac{R}{1+{\left(i\omega \tau \right)}^{\psi }}$$

where the parameter, ψ, admits values between zero and one.

In this work, an electrical circuit composed of an electrode resistance ($Z_{electrode}$) in series with two blocks that have resistances and capacitances of the grain regions ($R_{g}$ and $C_{g}$) and grain boundary regions ($R_{gb}$ and $C_{gb}$), where the complex impedance can be written by Equation (7):

(12) $$Z^{*}\left(\omega \right)=Z_{electrode}+Z_{g}^{*}+Z_{gb}^{*}$$

Therefore, considering the decentralization factor in both terms of the complex impedance for the grain region and grain boundary of Equation (11), we then have the following relationship for the complex impedance of the iron antimonate ceramic given by Equation (13):

(13) $$Z^{*}\left(\omega \right)=Z_{electrode}+\frac{R_{g}}{1+{\left(i\omega R_{g}C_{g}\right)}^{\psi }}+\frac{R_{gb}}{1+{\left(i\omega R_{gb}C_{gb}\right)}^{\psi }}$$

**Figure 8:**
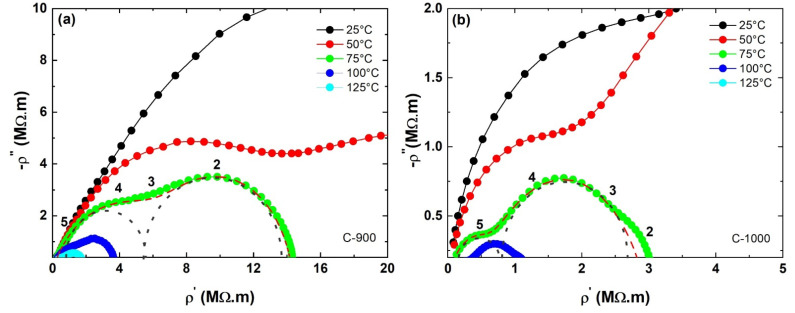
Argand diagram of iron antimonate ceramic pellets: (**a**) C-900; (**b**) C-1000. Numbers over the semicircles represent the power of the frequency. The dashed red line represents the adjustment performed on the semicircles of the grain region and grain boundary of the FeSbO_4_ and black dashed represents the deconvoluted grain and grain boundary regions.

Figure 8a,b shows the Argand or Nyquist diagrams (the complex plane) for the electrical impedance spectrum of the samples that presented a single phase (C-900 and C-1000). Electrical measurements were performed at temperature levels ranging from 25 to 250 °C. In this diagram, each experimental point was measured at a frequency that increases from right to left between values of 0.1 to $1\cdot 10^{6}\;\mathrm{Hz}$. Analyzing the impedance curves for a temperature of 75 °C, sample C-900 presented semicircles with higher values on the abscissa than sample C-1000. In both ceramic samples, the semicircles decrease with the increasing temperature of the FeSbO_4_ ceramic sample.

The resistances and capacitances of the grain regions ($R_{g}$ and $C_{g}$) and grain boundary ($R_{gb}$ and $C_{gb}$) were refined using the Zview software, version 2.9c, created by Dereck Johnson (North Carolina, USA) [23] that models equivalent circuits using experimental data from impedance spectroscopy. Furthermore, Figure 8 shows the Nyquist diagram of the samples studied in this work. The dashed red line represents the adjustment performed on the semicircles of the grain region and grain boundary of the FeSbO_4_ ceramic samples. In addition, the deconvoluted grain and grain boundary regions (black dashed lines) are indicated.

**Figure 9:**
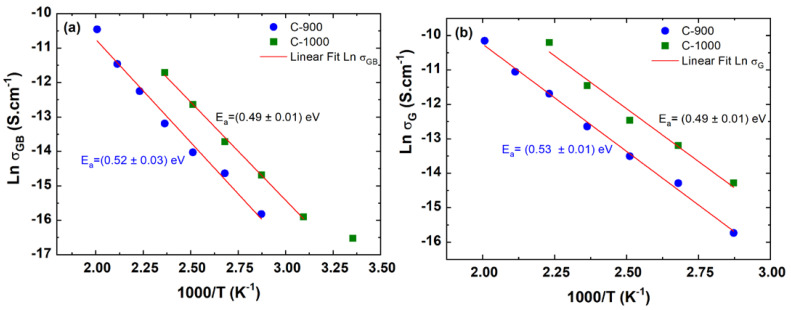
Arrhenius plots of the grain interior and grain boundary conductivities for FeSbO_4_ ceramics (C-900 and C-1000) for regions: (**a**) grain boundary; (**b**) grain.

From the adjustment of Equation (13) to the impedance spectroscopy experimental data, it was possible to determine the R and C values for the grain and grain boundary regions. To compare the electrical parameters, the resistances and capacitances were normalized by the geometric parameters (𝑑∙A^−1^) of the sample and to obtain the activation energy of the grain and grain boundary regions through the Arrhenius plot [27]; see Figure 9. To obtain the grain activation, and grain boundary energies, the Arrhenius equation for electrical conductivity was used, given by Equation (14):

(14) $$\sigma =\sigma_{0}exp^{\left(-\frac{E_{a}}{k_{B}T}\right)}$$

where $\sigma_{0}$ is the electrical conductivity associated with the charge carriers available for conduction in $S\cdot cm^{-1}$, $E_{a}$ is the activation energy required for the carrier to move in the crystal lattice of the ceramic in eV, $k_{B}$ is the Boltzmann constant in $eV\cdot K^{-1}$, and T is the temperature in K. To obtain the activation energy, $E_{a}$, it was necessary to perform a transformation of ln *σ* versus 1/𝑇, given by Equation (15):

(15) $$Ln\;\sigma =Ln\;\sigma_{0}-\frac{E_{a}}{k}\frac{1}{T}$$

Arrhenius plots of grain and grain boundary electrical conductivities for FeSbO_4_ ceramics are shown in Figure 9. Analyzing the Arrhenius graphs, it is possible to observe that the two ceramic samples of FeSbO_4_ presented single activation energy, responsible for the AC electrical conduction mechanism both for the grain region and grain boundary region. This statement is valid only for the temperature range studied in this work, which was between 25 to 250 °C.

**Figure 10:**
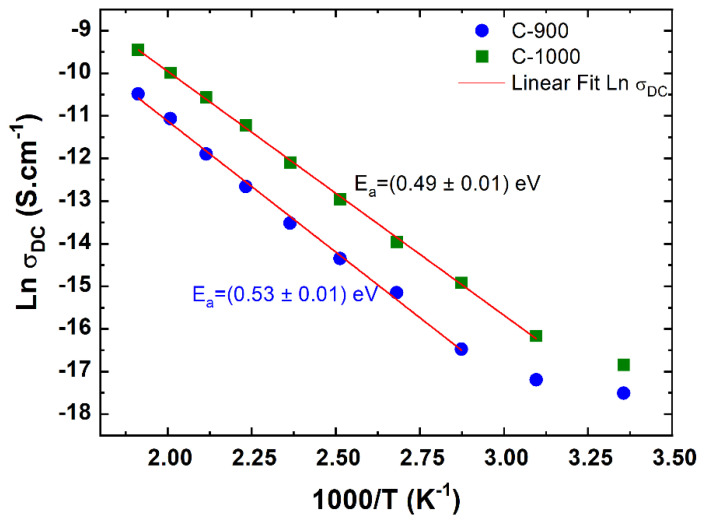
Variation of DC conductivity with the inverse of the temperature of ceramic samples, C-900 and C-1000.

Analyzing Figure 9b, it is possible to observe the influence of the calcination temperature (grain size) concerning the electrical conductivity of the grain, which is in orders of magnitude 2 from one sample to another. It is important to note that although the electrical conductivities of the grain boundary regions are smaller than the grain regions, their activation energies are very close, which is observed for both samples (Table 3). According to Mariappan et al. [32], even though the grain and grain boundary activation energy are virtually the same, they have a pre-exponential factor that differs considerably. Accordingly, in the grain boundary region, the blocking effect does not occur because of the high activation barriers but because of the geometric constriction effects. In addition to the activation energy involving AC electrical conduction processes, the activation energy at a frequency of 1 Hz was also determined, extrapolating as being a DC electrical conduction process; see Figure 10. It is possible to observe single activation energy responsible for the DC electrical conduction process measured at low frequencies in both iron antimonate ceramic samples.

For knowledge, there are few experimental articles in the literature regarding the study of the electrical and dielectric properties of FeSbO_4_ [3,4,5,6], particularly on the activation energy referring to the grain and grain boundary regions of the FeSbO_4_ ceramic pellet. Millet et al. [3] investigated the redox properties of pure V-doped FeSbO_4_ through electrical conductivity measurements of a ceramic powder under mechanical pressure of 10^5^ Pa, varying the temperature and with a controlled oxygen atmosphere. They determined the activation energy equal to 72.6 kJ∙mol^−1^ and equivalent to 0.75 eV [3]. Furthermore, Sing et al. [4] through producing thin films of pure FeSbO_4_, annealed at 450 °C for 2 h, and experimentally determined the activation energy around 0.49 eV from electrical resistance measurements as a function of temperature. Analyzing the results for the activation energy of the grain boundary region, as observed in Table 3, in the case of a porous ceramic sample (C-1000), activation energy was measured, $E_{a}=0.49\;\mathrm{eV}$, and an impact on its value was not observed to the detriment of the dense ceramic sample (C-900) with a value of $E_{a}=0.52\;\mathrm{eV}$. Therefore, a direct influence of the porosity (P = 36.8 %) contained in this ceramic sample on the activation energy of the grain boundary region is not observed. Comparing the activation energies of the grain region ($E_{a}=0.53$ and $E_{a}=0.49\;\mathrm{eV}$) of the samples, C-900 and C-1000, respectively, it was observed that the ceramics, C-900 with larger average grain size, D=3.79 μm, need a slightly higher activation energy to promote an increase in their AC electrical conductivity, corresponding to the contribution of the grain region. It can be concluded that despite the way of production of FeSbO_4_ ceramics, pressed and sintered ceramics are different in relation to other works, only pressed ceramics or thin films; the results for activation energy obtained here in this manuscript are in good agreement with both works published by [3,4].

**Figure 11:**
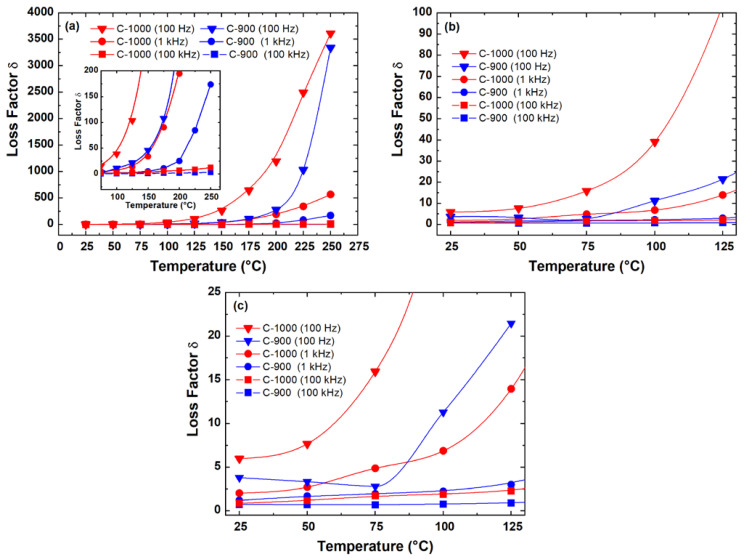
The temperature-dependent loss factor of FeSbO_4_ ceramics at (**a**) range of 25–250 °C; (**b**) zoomed between 20 to 130 °C; (**c**) zoomed in measurements from 20 to 130 °C, with recentering in loss factor values. All measurements were performed at 0.1, 1.0, and 100 kHz. The inset of (**a**) shows a zoom for a temperature between 75 and 260 °C.

Figure 11 illustrates the loss factor ($tan\;\delta =\frac{\varepsilon^{\prime \prime }}{\varepsilon^{\prime }}$) as a function of temperatures measured at 0.1, 1, and 100 kHz of the antimony ferrite samples. In the spectrum of Figure 11a an expected increase in the loss factor as a function of temperature can be observed in all samples. The inset is a zoom for a temperature between 75 and 260 °C to distinguish the temperature-dependent loss factor measured at 100 kHz for the C-900 and C-1000 samples. Figure 11b shows strong dispersion in loss factor at temperatures above 100 °C in the FeSbO_4_ ceramic samples measured at 0.1 kHz to measurements performed at frequencies of 1 and 100 kHz. The understanding of this high dispersion at low frequencies can be explained mainly by the relationship between the electrical conductivity and the loss factor given by $\sigma =\omega \varepsilon^{\prime }tan\delta$ where ω is the frequency and, $\varepsilon^{\prime }$ is the real permittivity [33]. As seen in Table 2 and Figure 11a, the porous ceramics (C-1000) have an increase in their conductivity due to the influence of pores in the grain boundary region; therefore, there was an increase in their loss factor, δ. Emphasizing that this high dispersion at low frequencies may also have a smaller contribution as a function of grain size.

Considering that at high frequencies, there will only be the influence or contribution of the FeSbO_4_ ceramic grain region, it is possible to observe in Figure 11c and Table 4 that the loss factor at the frequency of 100 kHz showed a slight increase as a function of the decrease of the average grain size. This hypothesis can be validated from an analysis of the location of the frequency equal to 10^5^ Hz, as an example, in the complex impedance measurements shown in Figure 8, performed at a temperature of 75 °C.

The sample C-1000 with a smaller grain size, $\underline{D}=2.53\;\mu m$, showed a higher loss factor in the entire temperature range compared to the sample, C-900, with $\underline{D}=3.79\;\mu m$. Analyzing the data contained in Figure 11b, at a temperature of 25 °C, it was observed that the C-900 ceramic presented a loss factor equal to 1.20 and the C-1000 sample a factor equal to 2.03, at f = 1 kHz. However, at f=100 Hz, an increase in the loss factor was observed where samples C-900 and C-1000, respectively, presented values equal to 3.8 and 5.99, see Table 4. It is concluded that the loss factor results corroborate the measurements of electrical conductivity, dielectric constant, and activation energy obtained in this work.

## 4. Conclusions

In this work, FeSbO_4_ powder was prepared using the solid-state reaction method. Afterward, dense and porous ceramics were obtained by sintering the pressed powder at temperatures of 900 and 1000 °C. Rietveld profile analysis of the powder X-ray diffraction data showed that FeSbO_4_ adopts the trirutile-type structure (space group *P*4_2_/*mnm* with a ≅ 4.63 Å and c ≅ 9.23 Å). The dielectric properties were measured in the frequency range of 10^−1^ Hz–1 MHz, as a function of temperature (25–250 °C). The real (σ′) and imaginary (σ″) parts of the complex conductivity increase with rising annealing temperature for both samples. The real conductivity in the AC region for 𝑓 = 100 kHz was $1.59\times 10^{-6}\;S\cdot {\mathrm{cm}}^{-1}$ and $7.04\times 10^{-7}\;S\cdot {\mathrm{cm}}^{-1}$ for the ceramic samples obtained from the powder calcined at 900 (C-900) and 1000 °C (C-1000), respectively. Furthermore, the dielectric constants (k′) measured at room temperature and f=100 kHz were 13.77 (C-900) and 6.27 (C-1000), while the activation energies of the grain region were E_a_ = 0.53 eV and E_a_ = 0.49 eV, respectively. Similar activation energy (E_a_ = 0.52 eV and 0.49 eV) was also obtained by the brick-layer model and confirmed by the adjustment of activation energy by DC measurements, which indicated an absence of the porosity influence on the parameter. Additionally, loss factor values equal to 3.8 (C-900) and 5.99 (C-1000) were obtained for measurements performed at 100 Hz, suggesting a contribution of the conductivity originated from the combination or accommodation of the pores in the grain boundary region. Thus, our results prove that the microstructural factors that play a critical role in the electrical and dielectric properties are the average grain size and the porosity interspersed with the grain boundary region.

## Figures and Tables

**Table 1:** Crystal structure data of FeSbO_4_ as refined from X-ray powder diffraction data for the powder calcined at 900 and 1000 °C.

Empirical Formula		FeSbO_4_	FeSbO_4_
Calcining temperature (°C)		900	1000
formula weight (g∙mol^−1^)		241.60	241.60
wavelength (Å)		1.54056	1.54056
crystal system		tetragonal	tetragonal
space group		*P*4_2_/*mnm*	*P*4_2_/*mnm*
*a = b* (Å)		4.63736(7)	4.63438(2)
*c* (Å)		9.23042(1)	9.21919(4)
α = β = γ (deg)		90	90
volume (Å^3^)		198.50(1)	198.01(1)
density _calc_, (g∙cm^−3^)		6.098	5.552
Z		2	2
reflections collected		20°< 2θ < 80°	20°< 2θ < 80°
χ^2^		1.96	1.12
Bragg factor R_B_ (%)		2.85	2.56
R_F_ factor (%)		2.98	2.66
atoms		Wyckoff sites and atom coordinates	
Sb1		2a	2a
	*x = y = z*	0	0
Sb2		2a	2a
	*x = y = z*	1/2	1/2
Fe1		4e	4e
	*x = y*	0	0
	*z*	0.33884(1)	0.33417(1)
Fe2		4e	4e
	*x = y*	1/2	1/2
	*z*	0.13187(2)	0.17695(6)
(Sb/Fe)3		4e	4e
	*x = y*	0	0
	*z*	0.32179(2)	0.33797(4)
(Sb/Fe)4		4e	4e
	*x = y*	1/2	1/2
	*z*	0.83324(2)	0.83312(2)
O1		4f	4f
	*x = y*	0.3467(1)	0.2973(8)
	*z*	0	0
O2		8j	8j
	*x = y*	0.3032(9)	0.3076(5)
	*z*	0.3792(5)	0.3251(7)

**Table 2:** Dielectric constant and real electrical conductivity measurements of ceramic samples C-900 and C-1000 performed at 10 Hz and/or 100 kHz at room temperature.

Sample	$\sigma^{\prime }$		$k^{\prime }$	$\underline{D}$ **(μm)**
	f = 10 Hz	f= 100 kHz	f= 100 kHz	
C-900	2.95 × 10^−8^	1.59 × 10^−6^	13.77	3.79 ± 1.06
C-1000	5.51 × 10^−8^	7.04 × 10^−7^	6.27	2.53 ± 0.68

**Table 3:** Grain and grain boundary activation energy obtained by BLM and DC conductivity for C-900 and C-1000 ceramics.

	Activation Energy by Brick-Layer Model		
**Sample**	**Ea ± ΔEa (Grain Boundary) (eV)**	**Ea ± ΔEa (Grain) (eV)**	$\underline{D}$ **(μm)**
C-900	0.52 ± 0.03	0.53 ± 0.01	3.79 ± 1.06
C-1000	0.49 ± 0.01	0.49 ± 0.01	2.53 ± 0.68
	**Activation Energy for DC conductivity (f = 1 Hz)**		
	**Ea ± ΔEa (eV)**		$\underline{D}$ **(μm)**
C-900	0.53 ± 0.01		3.79 ± 1.06
C-1000	0.49 ± 0.01		2.53 ± 0.68

**Table 4:** Loss factor measurements of ceramic samples, C-900 and C-1000, performed at 100 Hz, 1 kHz, and 100 kHz, at room temperature (25 °C).

Sample	tan δ			$\underline{D}$ **(μm)**
	f = 100 Hz	f = 1 kHz	f = 100 Hz	
C-900	3.80	1.20	0.75	3.79 ± 1.06
C-1000	5.99	2.03	0.86	2.53 ± 0.68

## Data Availability

Not applicable.
